# Chloroplast genome structure and phylogeny of *Spiranthes sinensis*, an endangered medicinal orchid plant

**DOI:** 10.1080/23802359.2019.1664345

**Published:** 2019-09-13

**Authors:** Jing Fan, Ming-Yuan Huang

**Affiliations:** College of Life Sciences, Leshan Normal University, Leshan, P.R. China

**Keywords:** *Spiranthes sinensis*, complete chloroplast genome, orchid plant, Illumina sequencing

## Abstract

*Spiranthes sinensis* is an important medicinal plant of *Spiranthes* family and is currently in an endangered state. To better guide the systematic classification of *S. sinensis*, the complete genome of its chloroplast was sequenced and characterized. The complete chloroplast genome is 152,786 bp in length and contains a large single copy (LSC) region of 83,446 bp, a small single copy (SSC) region of 17,938 bp, and two inverted repeat (IR_A_ and IR_B_) regions of 25,701 bp. The genome encodes 132 genes (112 unique genes), including 86 protein-coding genes (78 unique genes), 8 rRNA genes (4 unique genes), and 38 tRNA genes (30 unique genes). The total GC content of plasmid genome is 36.18%. Phylogenetic results indicated that *S. sinensis* is more closely related to *Ludisia discolor*, *Goodyera schlechtendaliana*, *Goodyera fumata*, and *Goodyera procera*. The study enriches the plasmid genomic information of *S. sinensis* and is of great significance for the genetic protection of this species.

*Spiranthes sinensis* is a well-known Chinese herb in the genus *Spiranthes*. It is widely used in the treatment of inflammation, anti-cancer, anti-diabetic, and other diseases (Peng et al. 2007; Gutiérrez 2010; Shie et al. 2015). In addition, due to the spiral inflorescence, this plant also has a high ornamental value. However, as a result of environmental damage and over-exploitation, the number of *S. sinensis* fell sharply and was already listed as a category II protected plant in china. *Spiranthes sinensis* is a small terrestrial plant that grows almost like weeds in lowlands, meadows, or hillsides. Due to the morphological polymorphism caused by natural hybridization and polyploidy, species delimitation in the genus *Spiranthes* has long been a problem (Dueck and Cameron 2007). Molecular systematics contributes to the classification of species. In most cases, molecular identification is carried out using a single or several genes, resulting in limited genetic information. The complete chloroplast genome has been widely used in plant species definition and phylogenetic evolution (Fan et al. 2019). However, there is currently no complete chloroplast genome sequence of *S. sinensis* in the NCBI database. This study aimed to decipher the chloroplast genomic information of *S. sinensis* and provide a basis for its genetic classification and systematic evolution.

The leaves of *S. sinensis* were collected from the germplasm resource nursery in Leshan, Sichuan Province, China (103°39′14ʺ E, 29°18′45ʺ N), the voucher specimen (SW0419) was deposited in the herbarium of Leshan normal university. Total genomic DNA was extracted by the SDS method, and the complete genome sequencing of chloroplast was carried out on the Illumina HiSeq Xten platform. The collected data were qualitatively controlled by NGSQC Toolkit v2.3.3 (Patel and Jain 2012), assembled using SPAdes v. 3.11.0 (Bankevich et al. 2012) and annotated with Plann software (Huang and Cronk 2015). The total chloroplast genome size of *S. sinensis is* 152,786 bp (GenBank accession number MK936427), in which the large single-copy region (LSC) is 83,446 bp, the small single-copy region (SSC) is 17,938 bp, and two inverted repeat sequences are 25,701 bp. A total of 132 genes were annotated, including 86 protein-coded genes, 8 rRNA genes, and 38 tRNA genes. Of which 112 genes are unique, 1 rps12 gene has trans-splicing, 14 genes contain an intron, and 2 genes contain two introns.

To determine the phylogenetic status of *S. sinensis*, 38 complete chloroplast genomes were aligned with the automatic alignment model of MAFFT 7.037 (Katoh and Standley 2013). Then, the best-fit nucleotide substitution model was calculated and the Maximum Likelihood (ML) phylogenetic tree was constructed by MEGAX software (Kumar et al. 2018). The parameters included 1000 bootstrap repetitions, general-time-reversible (GTR) nucleotide substitution model, Gamma distributed with Invariant sites (G + I), and complete deletion of gaps/missing data. Phylogenetic analysis showed that the chloroplast genome sequence of *S. sinensis* had a high resolution in molecular identification, which could distinguish *S. sinensis* from other plants (Figure 1). The complete chloroplast genome sequence decoded in this study provides important molecular data for molecular identification and genetic protection of *S. sinensis*.

**Figure 1.**
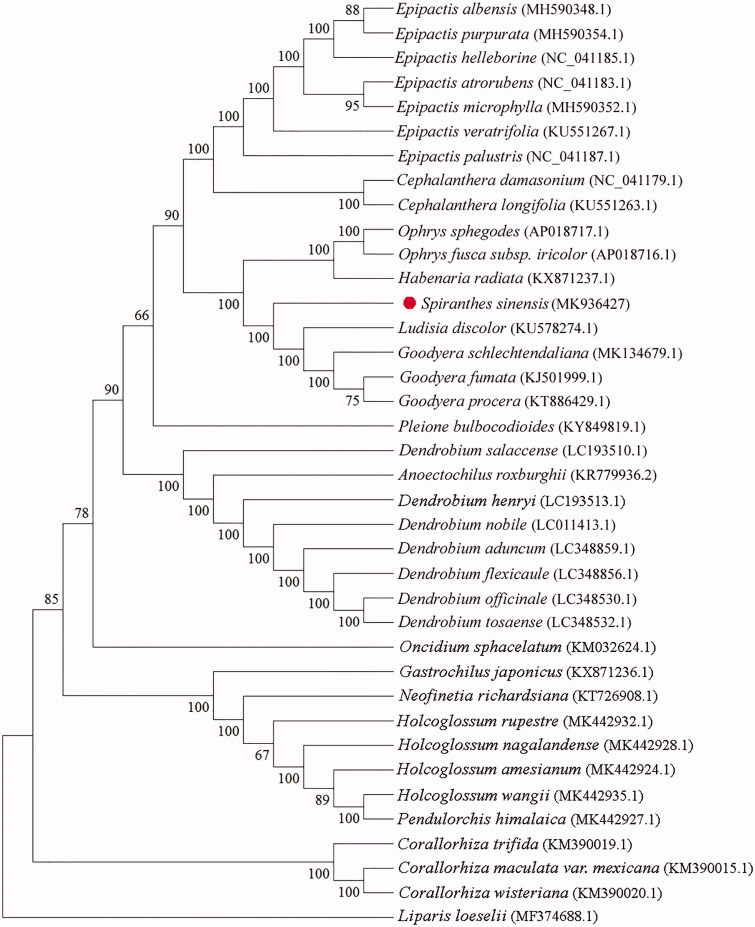
The maximum likelihood (ML) phylogenetic tree based on 38 chloroplast genome sequences was constructed by MEGAX software. Note: Numbers near each node represent the percentage values given by 1000 bootstrap analysis.
